# Identification of the Cohesive Parameters for Modelling of Bonded Joints between Flat Composite Adherends with Thick Layer of Adhesive

**DOI:** 10.3390/ma17194880

**Published:** 2024-10-04

**Authors:** Petr Bernardin, Frantisek Sedlacek, Josef Kozak, Ludmila Kucerova, Vaclava Lasova

**Affiliations:** Faculty of Mechanical Engineering, University of West Bohemia in Pilsen, Univerzitni 2732/8, 301 00 Plzen, Czech Republic; berny@fst.zcu.cz (P.B.); skal@fst.zcu.cz (L.K.); lasova@fst.zcu.cz (V.L.)

**Keywords:** bonded joint, composite, finite element, Scotch-Weld DP490, damage modeling, identification

## Abstract

The failure of bonded composite materials is accompanied by specific failure modes. These are specifically Mode I, Mode II, Mode III, and their combination (so-called mixed mode). These modes depend on the direction and type of loading. The mechanical properties describing the damage initiation and the damage evolution are unique according to the type of adhesive and present mode of failure. However, a few research studies have focused on an adhesive thicknesses greater than 0.2 mm. The main objective of this research is to investigate the mechanical properties of a bonded joint with large adhesive thickness loaded according to Modes I and II. The observed failure parameters, the cohesive and damage parameters, are identified by minimizing the difference between the force–displacement diagram obtained from the experimental data for both Mode I and Mode II. The finite element model is confronted with these parameters and is evaluated based on their agreement. Compared to other studies with a small adhesive layer thickness, the values of failure parameters are lower. The results show that the adhesive thickness has an influence on the values of cohesive and damage parameters and that these parameter values decrease significantly compared to a small adhesive thickness. The obtained parameters can be further used to predict the fracture toughness of other bonded joints loaded in any direction.

## 1. Introduction

Composite materials together represent a very broad term that can cover large numbers and types of materials that have been commonly used in industry for several years. In general, they are materials composed of two or more phases whose properties are better than those of the individual phases alone. One phase serves as the binder that binds and holds the resulting material together and is called the matrix. The second phase serves to transfer the load and is usually referred to as the filler [1].

For the purposes of this study the definition of composite materials, was used similarly to the one used in the book by Harris [2]. Composite materials are meant to be fiber composites. Fiber composites are materials whose matrix is in the form of polymer resins and the binder material is long carbon, glass, silicon fiber, or another suitable material. In these materials, commonly called fiber-reinforced plastics (FRPs), the desired properties are achieved by layering individual laminae on top of each other at different angles and by the appropriate choice of matrix or binder materials.

Today, composite materials are commonly used in many industries such as automotive, aerospace, healthcare, marine, and aviation [3,4,5,6,7]. Their diverse and widespread use has been aided by the development and research into new and better matrices, reinforcements, and new manufacturing technologies, as stated by Ngo [3] and Clyne and Hull [4].

Complex shapes of real engineering parts usually require joining. Joining two composite parts together is typically achieved via mechanical joints, bonded joints, fusion joints, and hybrid joints. Mechanical joints are joints in which the parts are connected by another member such as rivets, screws, and pins. Fusion joints include resistance welding, induction welding and ultrasonic welding. Bonded joints are joints in which two parts are joined by a relatively thin adhesive layer [8,9,10,11,12].

Malekinejad et al. [13] stated that adhesive bonded joints are superior to classical mechanical joints due to their lightweight and capability of reducing stress concentration. Pantelakis et al. [14] wrote very similarly about bonded joints. They stated that these joints reduce the cost, weight, and complexity of built construction, leading to the avoidance of fiber cuts and local stress concentrations. Adhesive bonded joints have also many disadvantages, as multiple authors have previously stated.

Bonding joints are affected by the contamination of bonded surfaces and environmental aging. They are also very affected by humidity and heat, and their strength depends on the strength of the material of adhesive layer, as stated by Pantelakis or Rezvaninasab [14,15]. This deficiency was investigated by Machado et al. [16]. Machado examined an experiment in which he used a mix of multiple adhesives for bonding composite carbon FPR laminates that resulted in increased static and impact strength.

Hybrid joints combine two types of joints listed above. An example would be a bonded joint with a bolt or rivet passing through the bonded layer. This element then acts as a local reinforcement to the joint and allows the bonded joint to carry more load [17,18,19]. In the work of Bodjona et al. [20], the effects of different adhesive thicknesses, the ductility of the bonded layer, and the effect of the hybrid joint on the initiation of the initial failure of the joint are compared. The influence of the screw in the bonded joint is found to be significant for bonded joints with high ductility and therefore high adhesive layer thickness.

In order to speed up the design of new products and make their introduction into operation faster and more reliable, it is necessary to know the relevant parameters for FEM analysis. These parameters are usually obtained from experiments, which will be described later.

There are many options for bonded joint modeling [21,22,23,24], differing by the dimension and size of used elements in the finite element (FE) model. The damage mechanics approach seems to give the most accurate prediction from crack initiation to failure.

The most common approach, which is also used in this study, is the cohesive model (CM) [25,26,27]. The CM describes cohesive stiffness, damage initiation, and damage evolution of the bonded joint. The CM is based on energy principles of linear elastic fracture mechanics, which has been developed since the beginning of 20th century [28,29,30]. This model is used for adhesive layers, where the macroscopic properties (Young’s modulus, Poisson’s ratio) of this layer disagrees with its real behavior [31]. These parameters strongly depend on the mode of failure, which can be detected during the damage evolution of the joint. This approach does not predict adhesive failure and mixed-mode failure. This is corrected by Ren’s and Li’s adhesive process zone model (APZM) [32]. In this model, the adhesive layer is considered as a 3D solid material. This model combines CM with the Cauchy–Born rule [33]. The Cauchy–Born rule is used to obtain the strain energy, which describes the bonding behavior at the molecular level within the adhesive layer. This behavior at the microscopic level leads to a nonlinear elastic response of the material at the macroscopic level. The deformation energy is converted to the elastic energy of the adhesive layer using the virtual internal bond (VIB) theory [34]. Thus, the material properties of the adhesive layer are obtained. Some input parameters for the Cauchy–Born rule have to be obtained experimentally. Ren and Li also mention in their study [32] that their model is more accurate than the classical CM and that it can correctly respond to a complex loading state. Even this model, however, cannot predict all possible modes of damage such as the formation of cracks at the interface between the adhesive and the composite parts. This can be simulated using models such as the coupled stress and energy criterion model [35]. Other authors approached the problem from a microscopic point of view and modeled the formation and propagation of the crack using only atomistic models [36,37].

**Figure 1:**
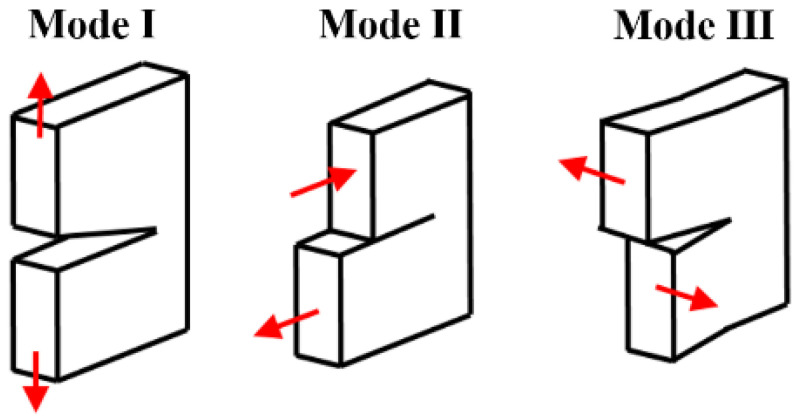
The description of the modes of failure.

Three failure modes are considered for the failure of composite materials in CM and are shown in Figure 1.

Each of these failure modes belongs to one of the main stress directions and can be described by the following three equations corresponding to each direction. The energy release rates $G_{I},G_{II},G_{III}$ of individual modes can be calculated according to the following equations:

(1) $$G_{I}=\frac{1}{2\Delta c}F_{22}\Delta y,$$

(2) $$G_{II}=\frac{1}{2\Delta c}F_{11}\Delta x,$$

(3) $$G_{III}=\frac{1}{2\Delta c}F_{33}\Delta z,$$

where $F_{11},F_{22},F_{33}$ are the reaction forces on the crack tip, Δx,Δy,Δz are the relative displacements of the fracture tip, and Δc is crack extension.

These modes are Mode I, Mode II, Mode III (described in Figure 1) or their mixed mode. The arbitrary mode of failure can be described as a superposition of three elementary modes of failure [38]. The cohesive and damage parameters are namely the critical strain energy release rate (SERR), effective separation, and maximal nominal stress. The value of the SERR for Modes I and II can be determined from experimental measurements according to the ASTM D5528 and ASTM D 7905 standards. To use composite materials in numerical simulations with any of the failure models described above, it is necessary to know the failure parameters listed above. For Mode I, an experiment called Double Cantilever Beam (DCB) can be used. This is a unilaterally stranded beam subjected to forces at its opposite end. An insert of the width of the test specimen is placed into the adhesive indentation. The energy required for Mode II crack propagation can be obtained by an experiment called End-Notch Flexure (ENF). This involves a simply supported beam loaded with a building force at the midpoint of its length. The magnitude of the force is controlled by the displacement. The test specimen again has an insert in the adhesion layer [39,40,41].

**Figure 2:**
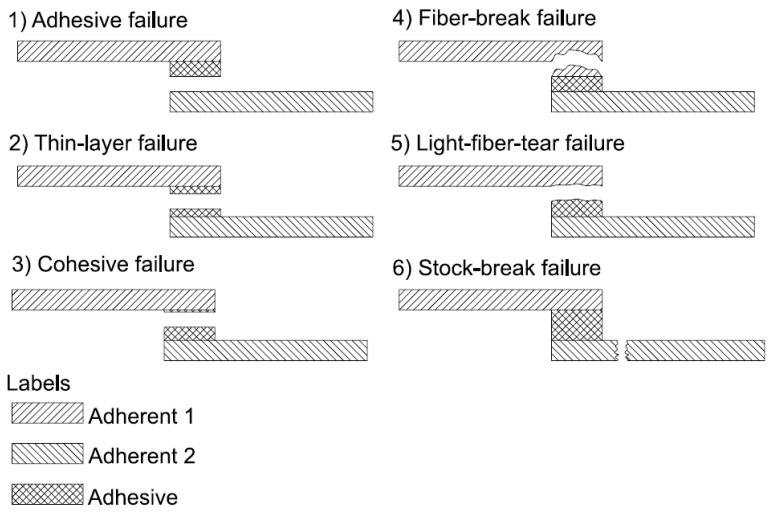
Failure mode of bonded FPR composite.

For bonded fiber composites in a resin matrix, six failure modes according to ASTM D5573 exist: adhesive failure, cohesive failure, thin-layer cohesive failure, fiber-tear failure, light-fiber-tear failure, stock-break failure, and mixed failure. These failure modes are shown in Figure 2. Other important failure modes in FRP materials are, for example, buckling and local delamination [42,43].

In [44] Kalina et al., the cohesive failure parameters of a composite woven material bonded with 3M Scotch-Weld DP490 adhesive were investigated. The bonded layer in this case had a thickness of 0.11 mm. In this work, only the failure parameters for Mode I were investigated. These results show that the numerical results correspond to the experimental results rather than the analytical ones, which do not consider the complex loading method of the cohesive layer. The value of the strain energy release for Mod I was around 900 J/m^2^. Experimental tests of failure and determination of cohesive and damage parameters for a unidirectional composite material bonded with different types of adhesives were performed in Kottner et al. [45]. The adhesives used were Huntsman Araldite 2021 and Gurit Spabond 345. In this case, a unidirectional composite was bonded, and the energy release rate values for Modes I and II were determined.

The cohesive zone model (CZM) model was used in the study by Faria and Campilho [46] for a numerical analysis of bonded joints of aluminum tubes using several types of adhesives. The tubes were loaded by tensile force. The results of these experiments and simulations are not similar to the results reported in this study because different adhesive types, specimens, and loading methods were used. The research by Ghabezi and Farahani [47] considered the development of a new traction-separation law model that would include both cohesive behavior and the fiber bridging phenomenon. This model was experimentally tested on samples containing Al_2_O_3_ nanoparticles. In the experimental samples in this work, only adhesive failure was present, and the bringing phenomenon was not observed.

The aim of this work was to find the cohesive and damage parameters for Mode I and Mode II for the adhesive Scotch-Weld DP490 applied on unidirectional carbon composite plates using previously mentioned ASTM test methods.

This was achieved by first experimentally testing the specimens and obtaining force–displacement curves. Furthermore, a numerical model was created, into which the CM of the adhesive layer was implemented. The force–displacement relationship was then also obtained from this calculation. The cohesive and damage parameters (e.g., *δ*_i_, *t*_i_, *G*_i_, *k*_i_) were varied until the minimum acceptable deviation of both relationships (force–displacement relationship from numerical simulation and from experiment) was obtained. Thus, the final values of cohesive and damage parameters were found for a specific composite material with a specific type of adhesive. Cohesive and damage parameters are influenced by many properties of the bonded joint itself (thickness, temperature, humidity, used material, etc.). The influence of the thickness of the adhesive layer was verified by comparing the mentioned parameters with the article [44], which was focused on the same adhesive and the same composite material used in the bonded joint. The use of a greater thickness of adhesive layer may result in a reduction in the stiffness of the whole bonded joint.

## 2. Experiment

**Figure 3:**
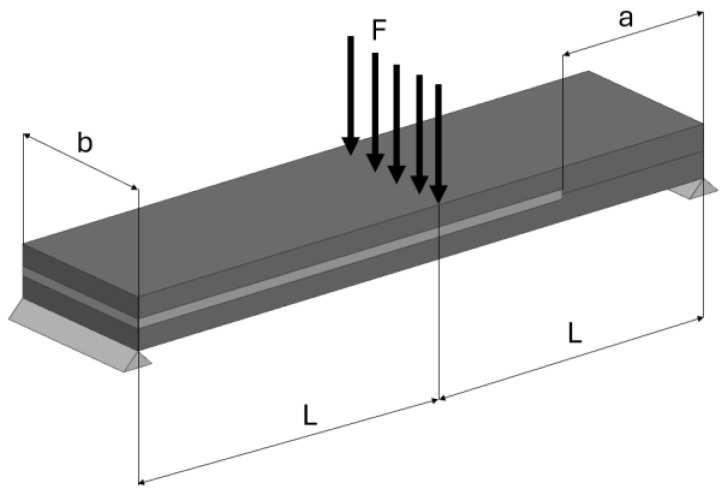
Arrangement of the ENF specimen.

The Double Cantilever Beam (DCB) test induces failure of the bonded joints according to Mode I. The End-Notched Flexure (ENF) test induces failure of the joints according to Mode II. Unidirectional carbon fiber composite Tenax HTS 5631 (sourced from Compotech, Susice, Czech Republic) strips were connected using the Scotch-Weld DP490 adhesive for both methods. This adhesive has a relative density of 1 and a shear strength of 30.2 N/mm^2^ at 23 °C. The arrangement of the ENF specimen is shown in Figure 3, described as follows: *b*—width of specimen; *a*—specimen length without an adhesive; *L*—half-length of the specimens; *F* -applied force.

**Figure 4:**
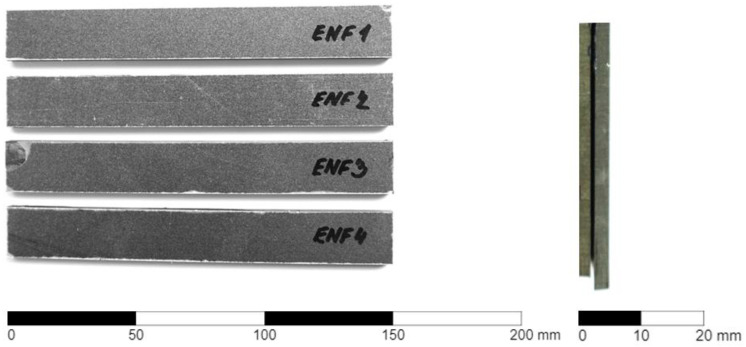
Manufactured specimens of type ENF (Right—four experimental specimens with corresponding numbers. Left—thickness of one specimen).

The ENF-type samples created for the experimental measurement of the first failure mode are shown in Figure 4.

**Figure 5:**
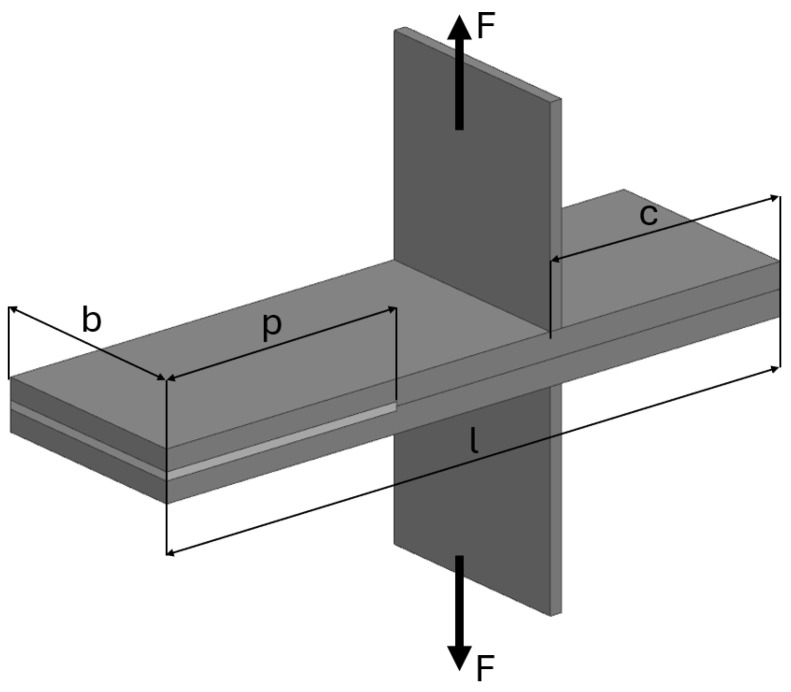
Arrangement of the DCB specimen.

The arrangement of the DCB specimen is shown in Figure 5. The standardized ASTM D5528 experiment for DCB specimens is defined as follows: *b*—width of specimen; *p*—length of cohesive layer; *l*—length of the specimens, *F* – applied force.

**Figure 6:**
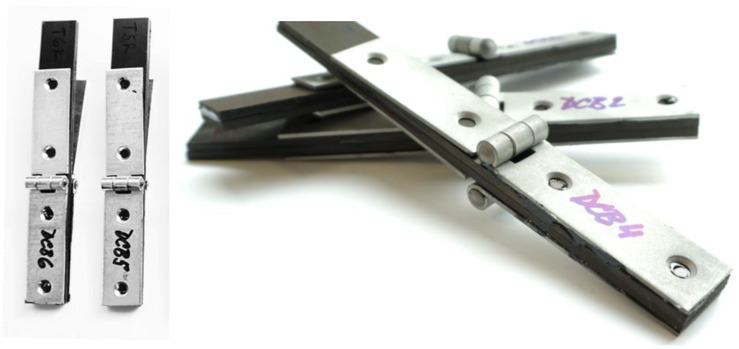
Manufactured specimens of type DCB.

The DCB-type samples created for the experimental measurement of the first failure mode are shown in Figure 6.

Unidirectional carbon fiber composite Tenax HTS 5631 strips were connected using the Scotch-Weld DP490 adhesive for both methods. The Dimensions column in Table 1 describes the geometry of the specimens, where t represents the thickness of specimen and *t*_adhesive_ is the thickness of the adhesive layer.

The adhesive was applied by hand without the use of fixtures. This achieved a higher thickness of the adhesive layer, averaging a thickness of 0.23 mm with a deviation of ±0.018 for the DCB experiment and averaging a thickness of 0.24 mm with a deviation of ±0.010 for the ENF experiment.

Mechanical properties of the Tenax HTS 5631 composite are shown in Table 2, where *V*_m_ is the matrix volume fraction, *V*_f_ is the fiber volume fraction, *E* is the Young’s modulus, G is the shear modulus, *ν* is the Poisson’s ratio, and indices 1, 2, 3 represent the specific direction. Six DCB specimens and four ENF specimens were experimentally tested. The Scotch-Weld DP490 adhesive was used.

**Figure 7:**
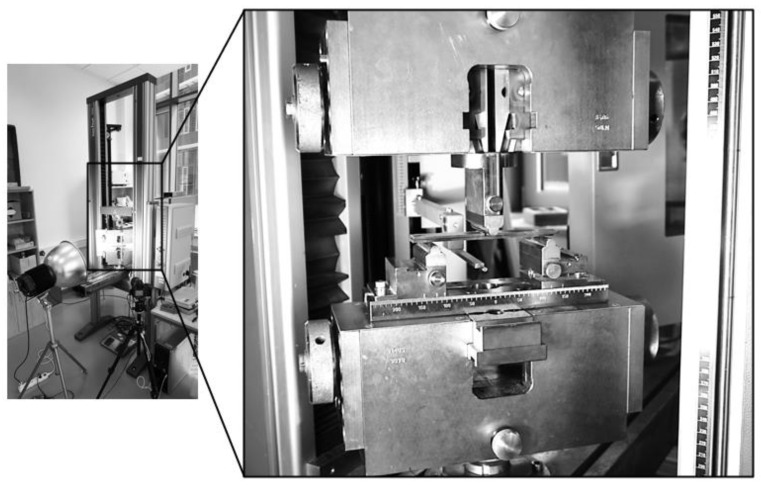
Zwick/Roell Z050 universal testing machine (**left**); details of specimen attachment (**right**).

The experiment was performed on the Zwick/Roell Z050 universal electro-mechanical testing machine with self-locking jaws and a 5 kN force cell (see Figure 7).

The experiment was performed at 23.2 °C, at a relative humidity of 62%. The loading was defined by the velocity *v*_y_ = 0.083 mm/s. The specimens were loaded until failure.

**Figure 8:**
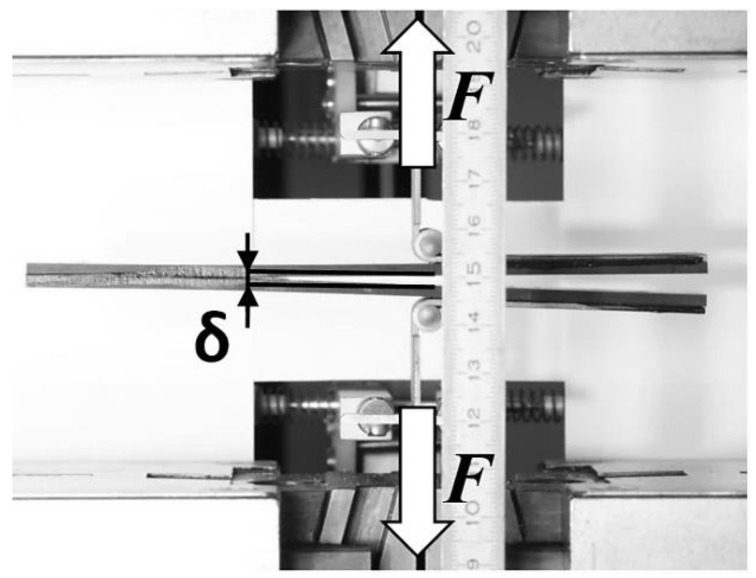
The DCB specimen testing using Zwick/Roell Z050.

**Figure 9:**
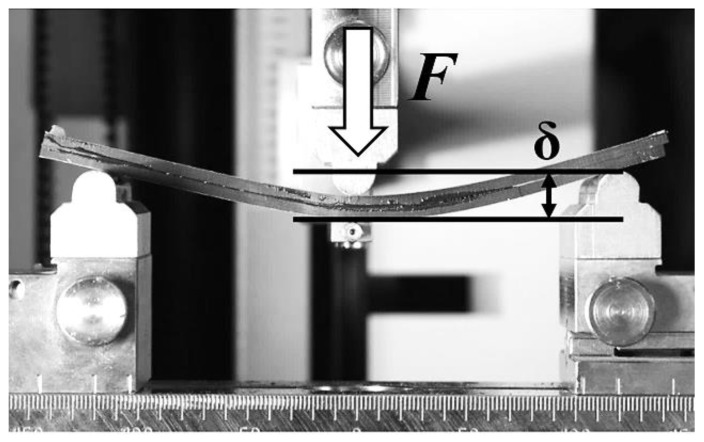
The ENF specimen testing using Zwick/Roell Z050.

Specimens were clamped on universal testing machine according to ASMT standards. The acting forces and the resulting displacement in the machine for Mode I and Mode II are shown in Figure 8 and Figure 9.

**Figure 10:**
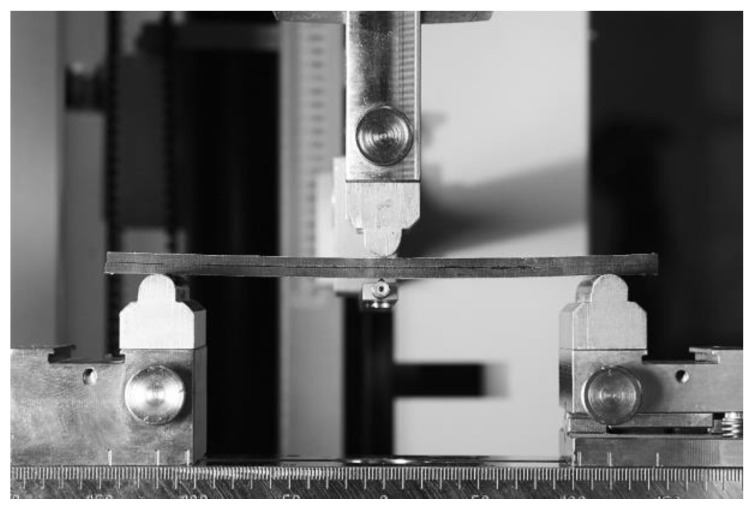
Start of the ENF experiment.

**Figure 11:**
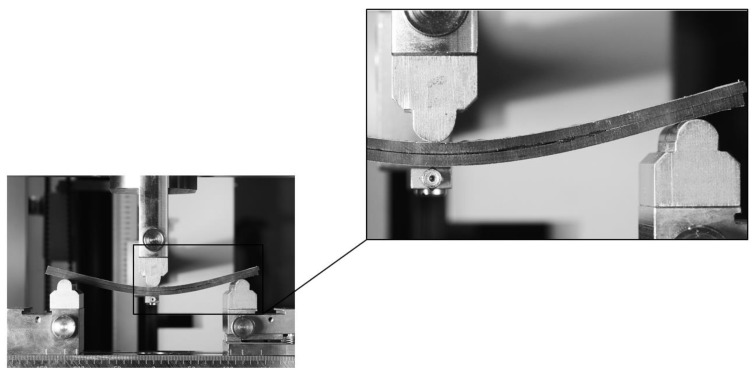
End of the ENF experiment with a failure on the right side of the figure.

**Figure 12:**
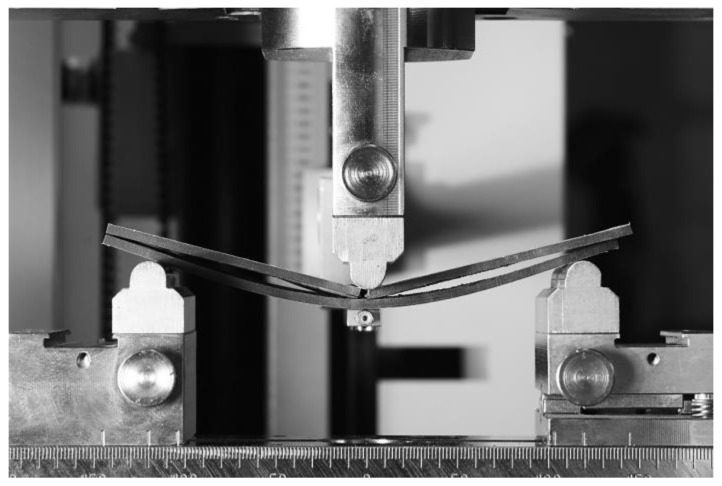
Break of the specimen during the ENF experiment.

The beginning of the ENF experiment for Mode II is show in Figure 10. Each specimen was clamped and loaded as described above. The failure of specimen 1 in the ENF experiment is shown in Figure 11. The failure of the adhesive part is visible on the right side of Figure 11. This failure is further propagated in the adhesive layer until the top composite part broke due to exceeding its strength limit, which can be seen in Figure 12. Additionally, the failure occurred as predicted and according to the ASTM standards.

**Figure 13:**
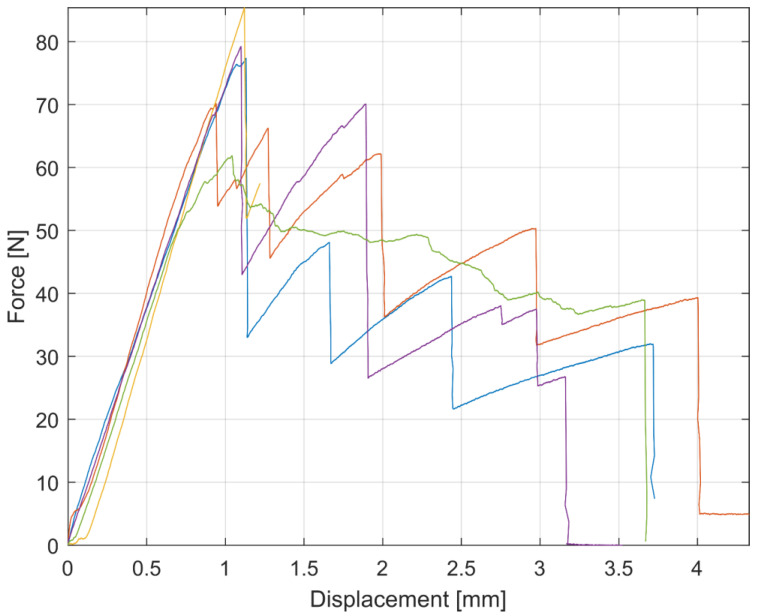
Force–displacement graph of experiment measurement of ENF specimens.

The force–displacement values from the ENF experiments for all specimens are plotted in Figure 13. The initial linear loading up to the maximum value and the subsequent decrease in force and its increase again can be seen in this figure. This behavior occurred several times and can be explained by the method in which the specimens are loaded and by the accumulation of the failure energy. During this loading, the force increases until all the accumulated failure energy is released into crack initiation or propagation. At crack initiation or propagation, the force decreases and the whole process repeats due to forced displacement loading of the machine jaws.

**Figure 14:**
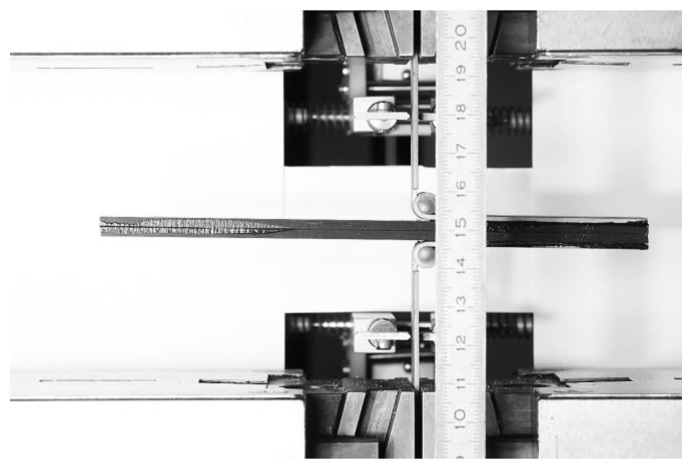
Start of the DCB experiment with specimen 3.

**Figure 15:**
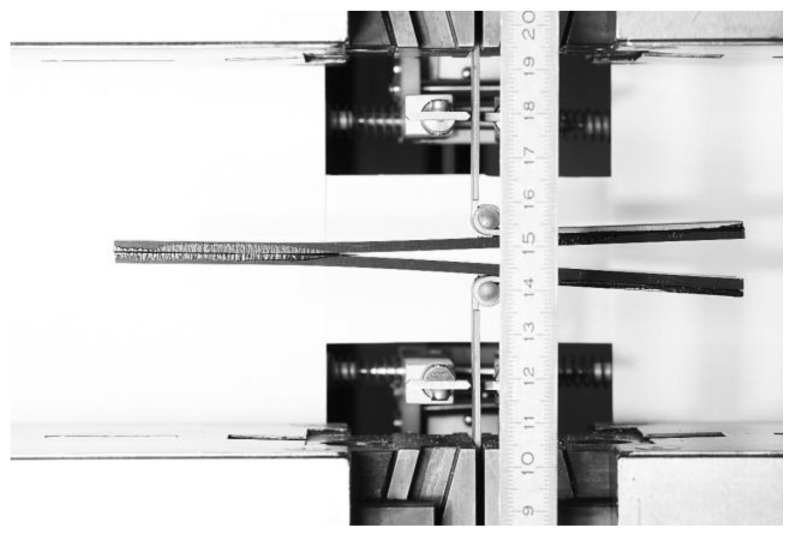
End of the DCB experiment with open left end.

**Figure 16:**
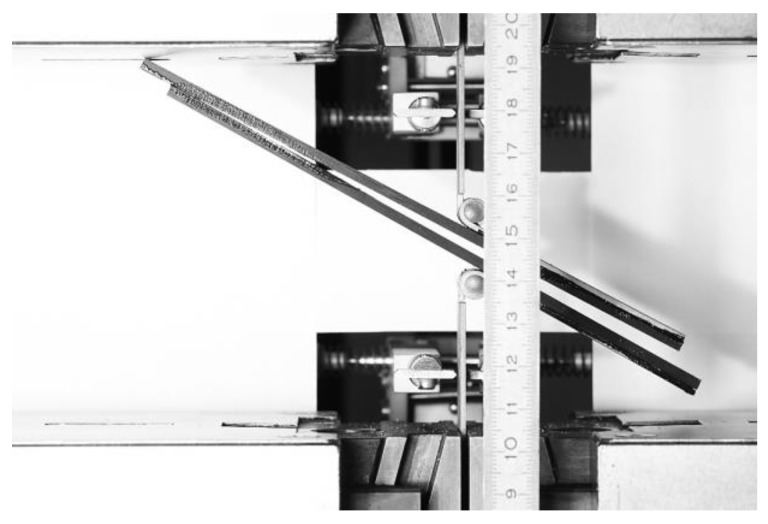
Collapse of the adhesive layer at the end of the DCB experiment.

The second experiment was performed according to the ASTM standard for DCB to obtain Mode I. Again, the specimens were clamped on universal testing machine and loaded until failure. The starting position of the machine with the undeformed specimen is shown in Figure 14. One end of the specimen starts to gradually open until total collapsed of the adhesive layer. The moment before the collapse is shown in Figure 15. The total collapsed is shown in Figure 16. Again, this is predicted behavior of the specimen in failure Mode I.

**Figure 17:**
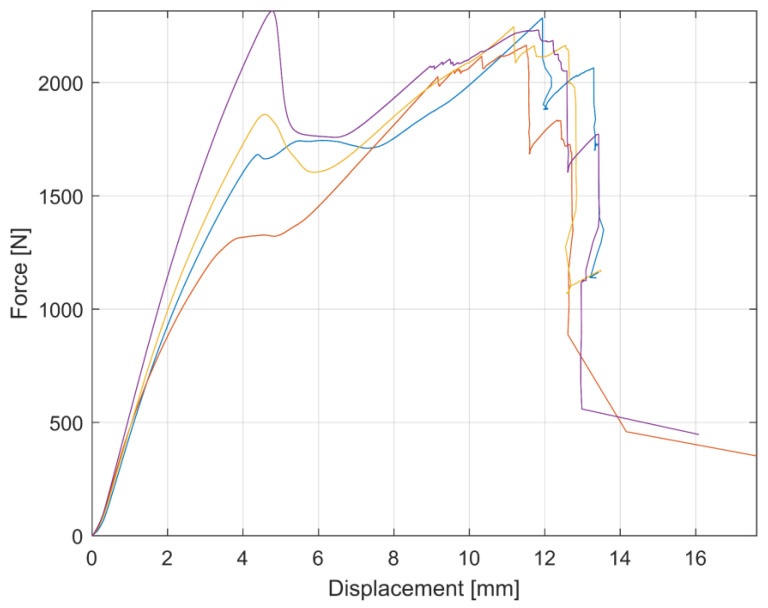
Force–displacement graph of experiment measurement of DCB specimens.

The force–displacement curves from the DCB experiments are shown in Figure 17. Similar behavior to the ENF experiment also occurred here. This can be explained by the accumulation of damage energy as in the case of ENF.

## 3. Finite Element Analysis

**Figure 18:**
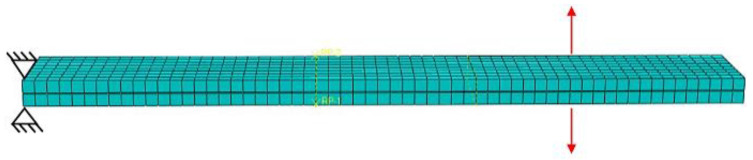
Three-dimensional meshed model of the DCB specimen (Red arrow—applied force).

**Figure 19:**
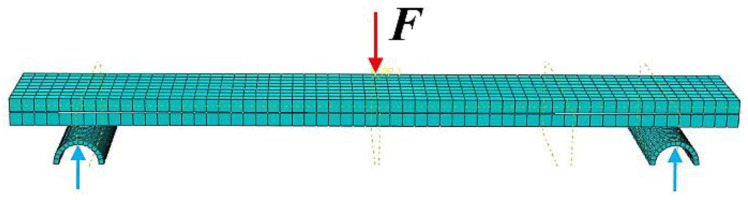
Three-dimensional meshed model of the ENF specimen (Red arrow—applied force, blue arrows—supports).

The FE model analysis of the experiments was performed using commercial software Abaqus/CAE 6.14. All parts were uniformly meshed with linear brick elements considering incompatible behavior. Material parameters were set for the composite material for both types of tests (DCB and ENF) in the FE model. The boundary conditions varied and were set to correspond with the DCB and ENF samples. Each plate in the model of DCB specimen was loaded by total displacement at specific nodes, according to Figure 18. In the model of the ENF specimen, touching contact was used between the deformable composite plate and the rigid cylindrical body. The loading displacement was applied at specific nodes, according to Figure 19. To simulate the adhesive coupling, the surface-to-surface cohesive (SSC) contact (with defined cohesive and damage parameters) was used.

**Figure 20:**
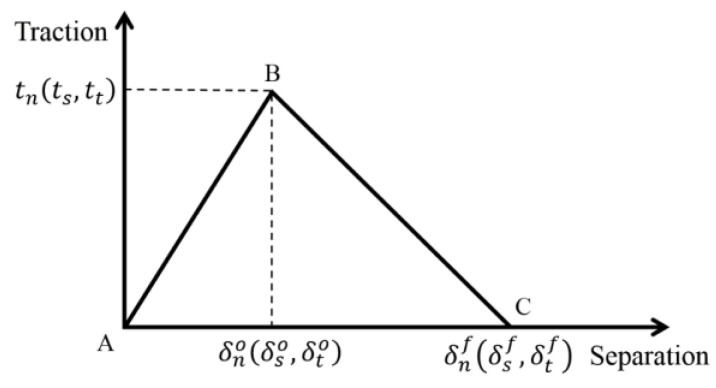
Linear damage parameters.

The behavior of the bonded joint can be idealized using the bi-linear CM (see Figure 20), where the slope of the AB curve represents the cohesive stiffness of the adhesives *K*_n_*, K*_s_*,* and *K*_t_, and n, s, and t represent the specific direction.

The slope of the BC curve is described by the damage initiation criterion and by damage evolution law [48] and refers to the softening process of the adhesive. In our case, the damage initiation was controlled by the maximum stress Equation (4).

(4) $$max\left\{\begin{array}{ccc} \frac{\langle t_{n}\rangle }{t_{n}^{o}}, & \frac{t_{s}}{t_{s}^{o}}, & \frac{t_{t}}{t_{t}^{o}} \end{array}\right\}=1$$

The denominator represents the maximum contact stress defined by the user ($t_{n}^{o},$ $t_{s}^{o}$, $t_{t}^{o}$) and the numerator represents traction stress. The index o represents the onset of damage propagation and f (see Figure 20) represents the state at complete failure of the bonded joint. The variable $\langle t_{n}\rangle$ is the normal traction stress in the Maxwell brackets, which describes the different behavior of the adhesive layer in positive and negative directions. The previously mentioned critical SERR is represented by the area under the two curves (AB, BC) in Figure 20.

## 4. Identification

The directions in the FE models of DCB and ENF specimens correspond with the directions of failure modes; namely, the n direction is equal to the direction of Mode I, the s direction is equal to the Mode II direction, and the t direction is equal to the Mode III direction.

The cohesive and damage parameters (*k*_i_, *t*_i_, *Gi*,) were set as input parameters and were found using the gradient optimization method implemented in OptiSLang 3.2.0. software. The identification of cohesive and damage parameters was performed by minimization of the difference in the force–displacement relationship obtained from the numerical analysis and from the experiment (see Equation (5)).

(5) $$r_{g}=\sum_{i=0}^{n}\frac{{\left(F_{FEA}^{i}-F_{exp.}^{i}\right)}^{2}}{\underset{i}{\max}\left(F_{exp.}^{i}\right)}$$

$F_{exp.}^{i}$ is the force gained from the numerical analysis, and $F_{FEA}^{i}$ is the force gained from the experiment. The difference, denoted as *r_g_*, is an objective function and was observed at each point of the two curves. This is a model parameter that must be minimized.

The mechanical properties (cohesive and damage parameters) for Mode I (*k*_I_, $t_{I}^{o}$, *G*_I_) and Mode II (*k*_II_, $t_{II}^{o}$, *G*_II_) were determined, respectively, for both test samples (DCB and ENF tests) using the FEA models.

## 5. Results

**Figure 21:**
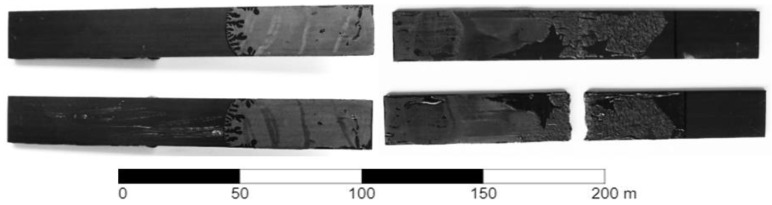
Specimens with appropriate mechanism of failure: DCB (**left**); ENF (**right**).

A series of experimental tests were performed on DCB and ENF specimens. Adherends were glued with a two-component epoxy-based 3M Scotch-Weld DP490 adhesive and loaded using the Zwick/Roell Z050 universal electromechanical testing machine with a 5 kN load cell. Tensile testing was performed under quasistatic load conditions (2 mm/min displacement rate). The failure of the adhesive was found in the DCB and ENF bonded joints with a cohesive character, instead of the pull-up of the fiber. The DCB and ENF ruptured specimens are shown in Figure 21.

**Figure 22:**
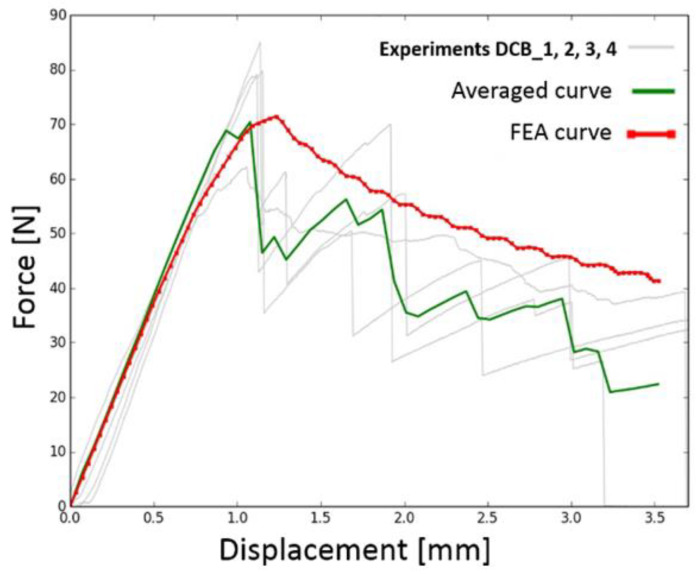
Comparison between tested specimens and approximation resulting curve for Mode I.

**Figure 23:**
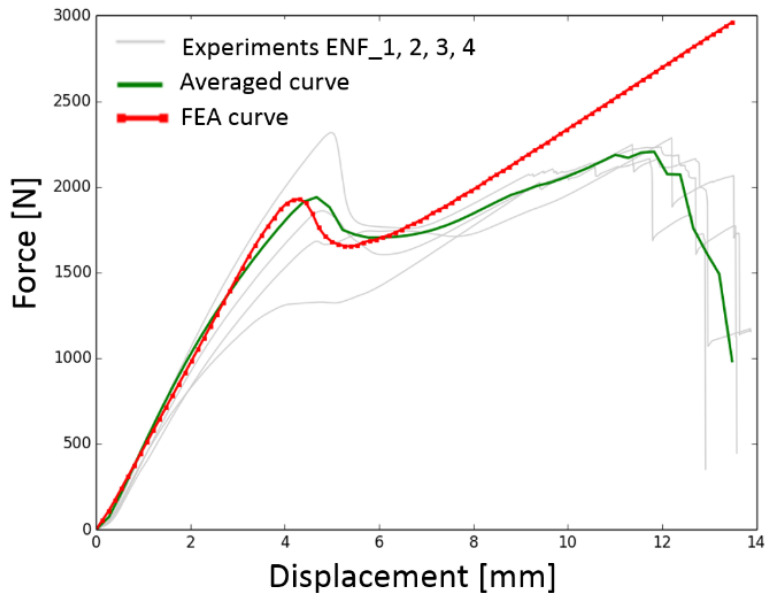
Comparison between tested specimens and resulting approximation curve for Mode II.

The relationships between force and displacement for all tested DCB and ENF specimens are shown in Figure 22 and Figure 23, where the curves obtained by the experiments are marked with a gray line, the averaged curve is marked by a solid line, and the fitted curve obtained from the FEA is marked by a dotted line.

The differences between the averaged values and the FEA values can be explained by the fact that the FEA model could not simulate the failure of the composite itself. This occurred in every specimen in the ENF experiment, as can be seen in Figure 23. This is plotted as a decrease in the experimental values on the right side of Figure 23. The possibility of failure of the composite specimen itself was not considered in the FEA model. This deviation of values, however, occurred in areas of large deformation, which are not important for the results of this study. The goal was to find the values of the first failure of the bonded joint, which is represented by the first oscillation in the plots in Figure 23.

## 6. Discussion

The critical SERR belonging to Modes I and II can be calculated directly from the experiment [49,50] using Equations (6) and (7).

(6) $$G_{Ic\;EXP}=\frac{3F_{c}\delta_{c}}{2b\left(a+\Delta \right)}$$

(7) $$G_{IIc\;EXP}=\frac{9{a^{2}F}_{c}\delta_{c}}{2b\left(2L^{3}+3a^{3}\right)},$$

Table 3 describes the manually obtained cohesive and damage parameters of the averaged curve for both testing methods (DCB and ENF), where the index i represents an appropriate mode of failure, while the used method of fitting the values of interface failure using numerical simulation shows a very good agreement. More precisely, from the point of view of Mode I, agreement was reached within 3% for the energy release rates (the numerical simulation reached the value *G*_I_ = 141.5 J/m^2^ vs. *G*_IEXP_ = 137.5 J/m^2^ from the experiment). And for Mode II, agreement was found within 4% (the numerical simulation reached the value *G*_II_ = 3848 J/m^2^ vs. *G*_IIEXP_ = 3983 J/m^2^ from the experiment). This stands out as a suitable method for determining the parameters for creating numerical models of glued joints using cohesive elements. All the interface parameters are listed in Table 3.

These parameters correlate with the values obtained experimentally and using FEM simulation with the chosen cohesive model. The other appropriate application of these parameters is for the detection of mixed modes. For these, the energy release rate in all three main fiber directions needs to be found. It is therefore necessary to obtain appropriate failure parameters for the third failure mode as well. These values can then be used for the FEM simulation of the composite mixed-mode failure. This is especially important for the design of shape-complex composite parts, where different failure modes can be manifested without the need to perform time-consuming and financially expensive complex experiments.

During the FEA simulations of the individual failure modes, not only were the same failure modes manifested during the breach, but also the failure mechanism was the same. During the simulation of the experiment according to the ENF method, shear deformations occurred in the bonded layer, which resulted in the displacement of the individual composite plates one after the other in the direction of Mode II failure. In the simulation of the DCB experiment, the bonded joint of the composite plates slowly opened up before the joint collapsed, similarly to the experiment described as above. Thus, there was also agreement with Mode I failure, as expected.

The values of the critical strain energy release rate in this case are six times lower than in the articles cited above [44,45]. These differences can be explained in several ways. The first is the thickness of the layer. This is a major contributor to the toughness of the bonded joint, and as the thickness of the adhesive layer increases, the toughness of the joint decreases. This is also evident from the stiffness of the adhesive itself, where in the case of [44], the stiffness is twice as high. Another influence contributing to the resulting critical value of the joint failure is the stiffness of the material used. This corresponds to the fiber direction of the unidirectional composite used in this case. In contrast, in the case of [44], a fabric was used which had similar values of Young’s modulus in two directions, but three times lower than Young’s modulus in the direction of fibers used in this article. The higher stiffness of the specimen put more stress on the adhesive layer and it tended to crack earlier, compared to the fabric in [44].

Different models of cohesive element behavior were used in comparison with [45], where an exponential model was used, versus the bilinear model used herein.

For Mode II, the critical strain energy release rate values are not so different. This can be explained by the fact that in this failure mode, the layers slide over each other and the Young’s modulus in the direction of the fibers does not play such a role here, nor does the thickness of the adhesive layer have an influence on its actual adhesive properties and cohesive properties at the adhesive–composite interface. This can also be seen in [45], where there is a large spread of *G*_II_ values compared to *G*_I_ values.

## 7. Conclusions

This study deals with the prediction of the failure behavior of bonded joints loaded according to a specific mode of failure. The main goal of this work was to determine unique mechanical parameters for failure Modes I and II for the Scotch-Weld DP490 adhesive. The identification of the mentioned parameters was performed by comparing the finite element analysis with the experiment. CM was used for the finite element analysis. The behavior of the bonded joint was described in this CM using the parameters SERR, cohesive stiffness, and maximum nominal stress. The experimental measurements were performed according to the ASTM standards using the DCB test (Mode I) and ENF test (Mode II).

- The critical strain energy release rate for Mode I was lower than in the other mentioned studies. This can be explained by the greater thickness of the adhesive and thus the lower stiffness of the bonded joint.
- For Mode II, the thickness of the adhesive layer was not as important for strain energy release rate *G*_II_, where the adhesive layers slide over each other more and the thickness of the adhesive layer did not have such a negative effect as in Mode I. So, the values were similar to those in other studies.
- The manually obtained cohesive and damage parameters for Mode I (*k*_I,_ $t_{I}^{o}$ *, G*_I_) and Mode II (*k*_II,_ $t_{II}^{o}$*, G*_II_) are valid for similar bonded joints loaded under the same mode of failure eventually under the combination of Modes I and II, using the 3M Scotch-Weld DP490 epoxy based adhesive.
- The values of SERR obtained by the FEA and the values calculated directly from experimental data according to ASTM D5528 and ASTM D 7905 [39,40] do not differ by more than 4%. It follows from this that the used fitting method is certainly applicable for these types of joints when it is necessary to obtain parameters for creating a correct numerical model.

## Figures and Tables

**Table 1:** Dimensions of experimental specimens.

Dimensions	DCB	ENF
*a* [mm]	35 ± 0.267	35 ± 0.200
*b* [mm]	20 ± 0.283	20 ± 0.225
*c* [mm]	60 ± 0.138	-
*l* [mm]	150 ± 0.250	-
*L* [mm]	-	75 ± 0.167
*t* [mm]	5 ± 0.100	5 ± 0.300
*t*_adhesive_ [mm]	0.21 ± 0.018	0.24 ± 0.010

**Table 2:** Material properties of composite plate.

*V*_m_		*V*_f_	
0.4		0.6	
*E*_1_[GPa]	*E*_2_[GPa]		*E*_3_[GPa]
100 (120)	8.00		8.00
*ν*_12_ [-]	*ν*_23_ [-]		*ν*_31_ [-]
0.370	0.315		0.022
*G*_12_[GPa]	*G*_23_[GPa]		*G*_31_[GPa]
4.00	3.04		4.00

**Table 3:** Cohesive and damage parameters of the bonded joints loaded under Modes I and II.

Parameters	i = I	i = II
*k*_i_ [GPa/m]	120	130
$t_{i}^{o}$ [MPa]	2.3	20.8
*G*_i_ [J/m^2^]	141.5	3848
*G*_i EXP_ [J/m^2^]	137.5	3983

## Data Availability

The original contributions presented in this study are included in the article, further inquiries can be directed to the corresponding authors.
